# High-Performance Potassium-Selective Biosensor Platform Based on Resistive Coupling of a-IGZO Coplanar-Gate Thin-Film Transistor

**DOI:** 10.3390/ijms24076164

**Published:** 2023-03-24

**Authors:** Tae-Hwan Hyun, Won-Ju Cho

**Affiliations:** Department of Electronic Materials Engineering, Kwangwoon University, 20, Gwangun-ro, Nowon-gu, Seoul 01897, Republic of Korea; gusxod97@kw.ac.kr

**Keywords:** potassium sensor, amorphous indium gallium zinc oxide, coplanar gate structure, extended gate, ion-sensitive field-effect transistor

## Abstract

The potassium (K^+^) ion is an essential mineral for balancing body fluids and electrolytes in biological systems and regulating bodily function. It is associated with various disorders. Given that it exists at a low concentration in the human body and should be maintained at a precisely stable level, the development of highly efficient potassium-selective sensors is attracting considerable interest in the healthcare field. Herein, we developed a high-performance, potassium-selective field-effect transistor-type biosensor platform based on an amorphous indium gallium zinc oxide coplanar-gate thin-film transistor using a resistive coupling effect with an extended gate containing a potassium-selective membrane. The proposed sensor can detect potassium in KCl solutions with a high sensitivity of 51.9 mV/dec while showing a low sensitivity of <6.6 mV/dec for NaCl, CaCl_2_, and pH buffer solutions, indicating its high selectivity to potassium. Self-amplification through the resistive-coupling effect enabled an even greater potassium sensitivity of 597.1 mV/dec. Additionally, we ensured the stability and reliability of short- and long-term detection through the assessment of non-ideal behaviors, including hysteresis and drift effects. Therefore, the proposed potassium-sensitive biosensor platform is applicable to high-performance detection in a living body, with high sensitivity and selectivity for potassium.

## 1. Introduction

Potassium is one of the most important biomarkers and is highly related to cellular activity, heartbeat, protein synthesis, carbohydrate metabolism, muscle motion, and the nervous system. In the human body, potassium accounts for approximately 4% of the mass, and approximately 98% of it exists inside cells, while the residual potassium flows in the extracellular blood serum. The concentration of potassium in the body is maintained at a level of 3.5–5.0 mM by the sodium/potassium-ATPase pump in the cell membrane [1,2,3]. A low potassium level is called hypokalemia, which is a common electrolyte disorder caused by the insufficient intake or consumption of potassium. It has been demonstrated that abnormal potassium levels (<2.5 mM) can cause high blood pressure, exhaustion, rhabdomyolysis, and an unusual heartbeat, which may lead to various illnesses such as stroke, arrhythmias, atrioventricular block, cardiac arrest, and even death in extreme cases [1,2,3,4,5]. Therefore, the accurate detection of potassium concentration is a major concern in the healthcare field for studying potassium-mediated physiological and pathological processes as well as to monitor potassium-related diseases. However, because potassium exists along with various physiological electrolyte ions, such as Ca^2+^, Na^+^, and H^+^, it is essential to selectively detect the precise concentration of potassium among different biological species in complex samples. Potassium sensors are widely applied in various fields, including water quality and soil monitoring, food hygiene, beverage production, pharmaceutical production, clinical applications, environmental protection, medicine, and healthcare. Moreover, extensive studies are being actively conducted on developing biosensors for the selective detection of potassium concentrations. Since Bergveld et al. first introduced the concept of a field-effect transistor (FET) that is sensitive to ions in the 1970s, there has been growing interest in applying the ion-sensitive field-effect transistor (ISFET) concept for converting chemical signals into electrical signals in various sensing applications [6]. In particular, ISFETs based on CMOS technology are very promising devices for sensing ionic activity in the human body because of their many beneficial features, such as their low cost, fast response time, small size, solid-state structure, easy signal processing, and label-free detection [7,8,9,10]. However, in conventional ISFETs, the polysilicon gate of a metal–oxide–semiconductor field-effect transistor (MOSFET) is removed, replaced by ion-sensitive membrane materials, and then immersed in target solutions to measure the electrochemical potential between the electrolytes and the sensing membrane due to the ion activity of the target solution. Therefore, ISFETs are sensitive to light and have low thermal stability, and the electrolyte leakage and chemical durability of ion-sensitive membranes determine their stability and reliability in biochemical sensor applications. In 1983, Spiegel et al. introduced the concept of an extended-gate field-effect transistor (EGFET) to replace the traditional ISFET, with advantages such as a low price, simple structure, easy packaging, long-term stability, light- and temperature-sensitivity, and disposable gate electrodes [11,12,13,14]. The ion-sensing membrane is separated from the gate of the MOSFET, and only the sensing membrane is immersed in the target solution to detect many target species, such as ions, chemicals, proteins, and viruses, while protecting the MOSFET from chemical damage [15,16,17,18,19,20]. However, conventional ISFETs, including EGFETs, despite their many advantages, have a fundamental limitation of sensitivity, referred to as the Nernstian limit, which severely restricts the commercial application of FET-type sensors. Therefore, enhancing the sensitivity beyond the Nernstian limit is the main objective for realizing high-performance sensor platforms [21,22,23,24]. Among the numerous approaches to overcome this physical limitation, multi-gate ISFETs, which replace traditional single-gate ISFETs, have attracted much attention. In particular, in a coplanar-gate structure ISFET, the gate electrodes are resistively coupled by resistive layers, resulting in the self-amplification of the sensitivity without the aid of an external amplifier circuit [25,26,27,28].

In this study, we propose a high-performance, potassium-selective FET-type biosensor platform based on the resistive coupling of thin-film transistors (TFTs). We introduced the concept of the EGFET, which avoids the shortcomings of traditional ISFETs and takes advantage of the disposable gate electrodes. A transducer unit based on an amorphous indium gallium zinc oxide (a-IGZO) coplanar-gate TFT, specifically designed for resistance coupling, and an EG-sensing unit were fabricated on separate glass substrates. The biosensor platform was constructed using an electrical connection between the two isolated units. The fundamental electrical characteristics, including the transfer and output characteristics of the resistively coupled transducer, were measured, and the self-amplification of the sensitivity by resistive coupling was estimated by means of resistance measurements of the series resistive layer of the coplanar-gate. The effectiveness of the sensitivity self-amplification by resistive coupling in the proposed sensing platform was verified by means of pH sensitivity measurements. The potassium-sensing properties, including sensitivity and selectivity, were evaluated using various solutions, such as pH, NaCl, CaCl_2_, and KCl buffer solutions. As a result, the proposed potassium-selective sensor demonstrateda higher selectivity for potassium than for sodium or calcium. In addition, the self-amplification by the resistive coupling effect reached 597.1 mV/dec, providing much greater potassium sensitivity. Through the evaluation of non-ideal behaviors, including hysteresis and drift effects, we also demonstrated the stability and reliability of the short- and long-term detection of potassium-selective biosensor platforms. Therefore, we expect the proposed potassium-selective sensor to be a promising biosensor platform with high sensitivity and reliability for selective detection of potassium in aqueous environments.

## 2. Results and Discussion

### 2.1. Electrical Characteristics of the Resistively Coupled TFT Transducer

**Figure 1:**
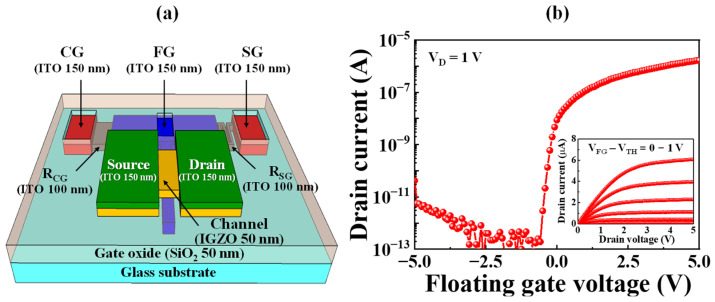
(**a**) Three-dimensional schematic of the fabricated resistively coupled TFT transducer unit. (**b**) Electrical characteristics of a-IGZO coplanar-gate TFT by FG operation. SG—sensing gate; CG—control gate; FG—floating gate; TFT—thin-film transistor.

Figure 1a shows a three-dimensional schematic of the fabricated resistively coupled a-IGZO coplanar-gate TFT transducer unit. The transducer unit was fabricated on a glass substrate with a thin a-IGZO channel layer where three coplanar gates and source and drain (S/D) electrodes were constructed with an indium tin oxide (ITO) film. Among the coplanar gates, the floating gate (FG) directly transfers the applied bias to the channel layer, the sensing gate (SG) transmits the surface potential of the sensing film on the sensing unit, and the control gate (CG) operates the FET. The CG and SG are connected in series to the FG via the resistive layers R_CG_ and R_SG_, respectively, thereby enabling the self-amplification of sensitivity in accordance with the resistance ratio (R_CG_:R_SG_). The sensing characteristics of FET-type sensor platforms are determined in terms of the shift in the transfer characteristic curve with a change in surface potential. Depending on the analyte concentration, the surface potential charge applied to the FET affects the threshold voltage, thereby shifting the transfer characteristic curve to the left or right depending on the positive and negative charges, respectively. Eventually, the FET-type sensor detects the concentration of biomolecules based on the magnitude of the voltage shift. Therefore, because the electrical characteristics of the transducer unit are important parameters closely related to the sensing characteristics of the entire sensor system, we evaluated the electrical characteristics of the prepared a-IGZO coplanar-gate TFT transducer by the FG operation, which directly transfers the bias to the TFT channel. Figure 1b shows the electrical characteristics of the FG operation, which directly transfers the surface potential of the EG-sensing membrane due to the analyte to the TFT channel. The transfer characteristic (I_D_-V_D_) curve was measured as an FG bias sweep between −5 and 5 V and at a drain voltage of 1 V. The output characteristic (I_D_-V_D_) curves of the inset were measured at 0.5 V intervals for the FG gate voltage from 0 to 5 V, while the drain voltage was swept from 0 to 5 V. The threshold voltage (V_TH_), field-effect mobility (μ_FE_), subthreshold swing (SS), and on/off current ratio (I_ON_/I_OFF_) extracted from the transfer characteristic curve were −0.3 V, 11.6 cm^2^/V·s, 128.1 mV/dec, and 2.6 × 10^7^, respectively, demonstrating the excellent performance of the proposed transducer device as a potassium-selective biosensor platform.

**Figure 2:**
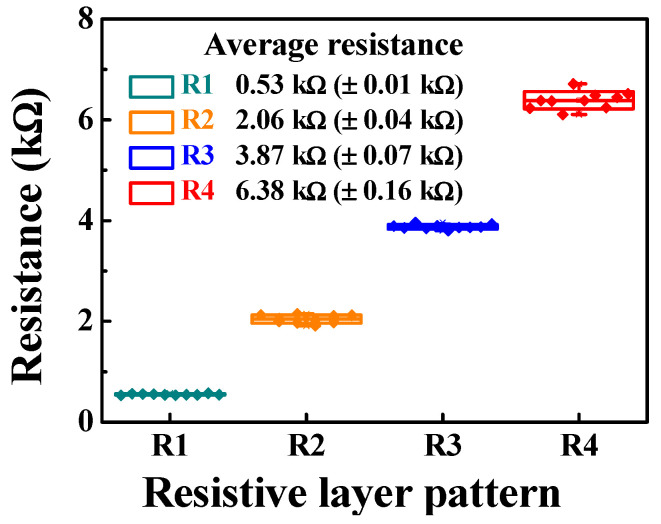
Resistance distribution extracted from the I–V characteristics, according to the resistive layer patterns.

Figure 2 shows the resistance distribution extracted from the I-V characteristics, according to the resistive layer pattern. To implement various R_CG_:R_SG_ resistance ratios of resistively coupled TFT transducers, the R_SG_ pattern was designed with four different sizes, whereas the R_CG_ pattern was designed to be the same size as the lowest R_SG_. It was found that the resistance measured from 10 resistors for each pattern (R1–R4) was uniform. The average resistances of R1, R2, R3, and R4 were 0.53, 2.06, 3.87, and 6.38 kΩ, respectively. Therefore, the R_CG_:R_SG_ ratios of the fabricated, resistively coupled TFT transducers were estimated to be 1:1, 4:1, 8:1, and 13:1, respectively. These results imply that the fabricated sensing platform is capable of flexible amplification of up to 13-fold its sensitivity through resistance-coupling-based self-amplification.

### 2.2. Self-Amplification of Low Sensitivity Based on Resistive Coupling

Conventional single-gate FET-type sensors have a physical sensitivity limit called the Nernstian limit. There is a need for an external amplification circuit to overcome this critical limitation in order to obtain high sensitivity. In this study, we designed an FET-type sensor with a coplanar-gate structure to enable the self-amplification of sensitivity based on the resistive-coupling effect between gate electrodes.

**Figure 3:**
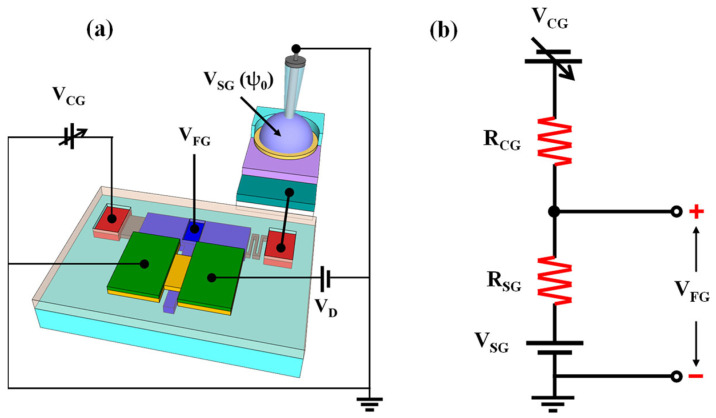
(**a**) Schematic diagram of the operation mode. (**b**) Simplified electrical equivalent circuit of the resistively coupled TFT transducer.

Figure 3a shows the electrical connection of the resistively coupled TFT transducer under self-amplifying operation in which the CG voltage (V_CG_) drives the transducer, while the SG voltage is connected to the sensing section to receive the surface potential (ψ_0_). Figure 3b shows a simplified electrical equivalent circuit that ignores external components in which the FG voltage (V_FG_) is connected in series with the V_CG_ and V_SG_. In this case, the relationship between V_FG_, V_CG_, and V_SG_ is expressed in terms of Equation (1). Moreover, V_CG_ can be reorganized into V_SG_, as expressed in Equation (2). Consequently, the relationship between the change in CG voltage (ΔV_CG_) and SG voltage (ΔV_SG_) can be expressed in terms of Equation (3). This result indicates that the small surface potential of the SG can be detected in the CG while being amplified by the magnitude of R_CG_:R_SG_. Therefore, the proposed biosensor platform can amplify the low sensitivity beyond the physical detection limit, according to the R_CG_:R_SG_ ratio, through resistive coupling.

(1) $$V_{\mathrm{FG}}=\frac{R_{\mathrm{SG}}}{R_{\mathrm{CG}}{+R}_{\mathrm{SG}}}V_{\mathrm{CG}}+\frac{R_{\mathrm{CG}}}{R_{T}}V_{\mathrm{SG}},$$

(2) $$V_{\mathrm{CG}}=\frac{R_{\mathrm{CG}}{+R}_{\mathrm{SG}}}{R_{\mathrm{SG}}}V_{\mathrm{FG}}-\frac{R_{\mathrm{CG}}}{R_{\mathrm{SG}}}V_{\mathrm{SG}},$$

(3) $$∴\;\Delta V_{\mathrm{CG}}\;\propto \;\frac{R_{\mathrm{CG}}}{R_{\mathrm{SG}}}\Delta V_{\mathrm{SG}},$$

### 2.3. pH-Sensing of the Resistively Coupled Coplanar-Gate EGFET Biosensors

**Figure 4:**
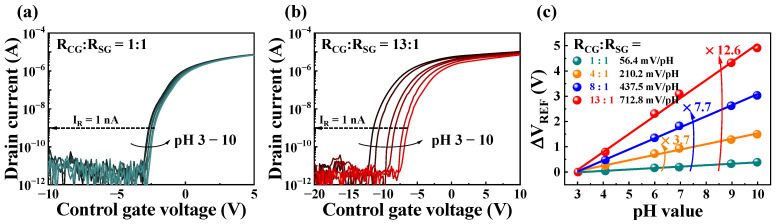
Transfer characteristic curves for R_CG_:R_SG_ ratios of 1:1 (**a**) and 13:1 (**b**) in pH buffer solution. (**c**) pH sensitivity at different R_CG_:R_SG_ ratios.

Prior to evaluating practical biomolecules, such as NaCl, CaCl_2_, and KCl, the self-amplification ratio at different resistance R_CG_:R_SG_ ratios was analyzed through pH-sensing measurements to validate their suitability for application in high-performance sensing platforms. Therefore, pH-sensing measurements were performed using a SnO_2_-sensing layer without a potassium-selective membrane. Figure 4 shows the pH-sensing characteristics of the FET-type biosensor, which consisted of a resistively coupled TFT transducer unit and SnO_2_ EG-sensing unit. Figure 4a,b show the typical transfer characteristic curves for R_CG_:R_SG_ ratios of 1:1 and 13:1, respectively. As the pH of the buffer solution increased (pH 3, 4, 6, 7, 9, and 10), there was a positive shift in the transfer characteristic curve. To quantify the shift of the transfer characteristic curve according to the pH of the buffer solution, we defined a drain current of 1 nA as the read current (I_R_) and the corresponding gate voltage as the reference voltage (V_REF_). Figure 4c shows ΔV_REF_ as a function of pH for different R_CG_:R_SG_ ratios. As the R_CG_:R_SG_ ratio increased from 1:1 to 4:1, 8:1, and 13:1, ΔV_REF_ showed linear increases of 3.7-, 7.7-, and 12.6-fold, respectively. Consequently, although the R_CG_:R_SG_ ratio of 1:1 resulted in a sensitivity of 56.4 mV/pH, the R_CG_:R_SG_ ratios of 4:1, 8:1, and 13:1 demonstrated high sensitivities of 210.2, 437.5, and 712.8 mV/pH, respectively, exceeding the theoretical Nernstian limit. Therefore, we verified that the proposed biosensor platform, which consists of a resistively coupled TFT transducer unit and an SnO_2_ EG-sensing unit, is a high-performance sensor capable of overcoming the limitations of existing FET-type sensors.

**Figure 5:**
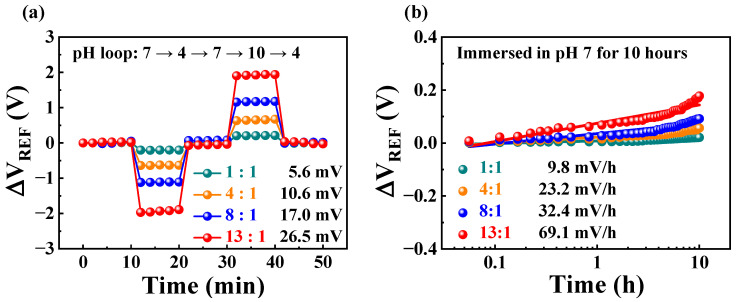
Non-ideal behaviors in pH-sensing operations. (**a**) Hysteresis and (**b**) drift effects at different R_CG_:R_SG_ ratios.

Applying the EG concept to an FET-type sensing platform protects the FETs from chemical damage. Nevertheless, chemical or physical reactions in the sensing membrane eventually lead to the degradation of sensing performance [29]. Therefore, it is necessary to verify the stability and reliability of high-performance sensing platforms by performing repetitive sensing tests. The stability and reliability of FET-type sensors are commonly evaluated by measuring non-ideal behaviors, such as hysteresis and drift effects [30,31,32]. The hysteresis effect of the constructed resistively coupled coplanar-gate EGFET sensor is illustrated in Figure 5a. While changing the buffer solutions in the order of pH 7, 4, 7, 9, and 7, the V_REF_ was measured five times at 2 min intervals in each solution, and the change was monitored. The hysteresis voltages (V_H_), defined as the difference between the initial and final ΔV_REF_, were 5.6, 10.6, 17.0, and 26.5 mV for R_CG_:R_SG_ ratios of 1:1, 4:1, 8:1, and 13:1, respectively. Figure 5b shows the drift effect measured after immersion in the pH 7 buffer solution for 10 h. The drift rates (R_D_) and ΔV_REF_ per unit time for R_CG_:R_SG_ ratios of 1:1, 4:1, 8:1, and 13:1 were 9.8, 23.2, 32.4, and 69.1 mV/h, respectively. The pH-sensing characteristics of the implemented resistively coupled coplanar-gate EGFET sensor are summarized in Table 1. Although V_H_ and R_D_ also increased as R_CG_:R_SG_ increased, the increase in V_H_ and R_D_ was small compared to the increase in sensitivity, demonstrating that there is little degradation of the pH-sensing characteristics in repetitive operation. Therefore, we consider the proposed resistively coupled coplanar-gate EGFET sensor suitable as a stable and reliable high-performance sensing platform.

### 2.4. Potassium-Selective Sensing by the Resistively Coupled Coplanar-Gate EGFET Biosensors

**Figure 6:**
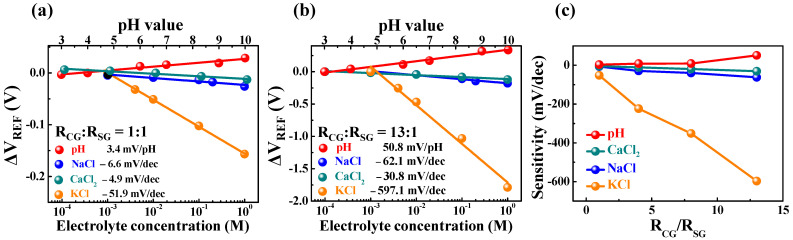
Reference voltage shift (ΔV_REF_) measured for pH, NaCl, CaCl_2_, and KCl solutions at R_CG_:R_SG_ ratios of (**a**) 1:1 and (**b**) 13:1. (**c**) Sensitivity for the pH, NaCl, CaCl_2_, and KCl solutions as a function of the R_CG_:R_SG_ ratio.

Potassium-selective FET-type biosensors were fabricated by drop-casting a potassium-selective membrane onto the SnO_2_-sensing layer of the EG-sensing unit. Valinomycin in the potassium-selective membrane serves as a potassium ionophore, permitting the transport of potassium only in the presence of various ions [33]. Therefore, the proposed potassium-selective sensor is capable of potassium-selective detection because only the surface potential of the sensing membrane generated by the transported potassium is transferred to the transducer unit SG. To verify the potassium-selective sensing behavior of the prepared device, the sensing characteristics in pH, NaCl, CaCl_2_, and KCl solutions were evaluated at various concentrations. Figure 6a,b show ΔV_REF_ as a function of the ion concentration in the prepared solutions for R_CG_:R_SG_ ratios of 1:1 and 13:1, respectively. The pH values of the buffer solutions were 3, 4, 6, 7, 9, and 10, while the concentrations of NaCl, CaCl_2_, and KCl solutions were 10^−4^, 10^−3^, 10^−2^, 10^−1^, and 10^0^ M. Figure 6c shows the sensitivity of the respective ions at different R_CG_:R_SG_ ratios, which increased as the R_CG_:R_SG_ ratio increased. In particular, an increase in sensitivity for potassium ions (K^+^) was noticeable, especially at the R_CG_:R_SG_ of 13:1 where it increased by 9.6-, 11.8-, and 19.4-fold compared to the sensitivity to the calcium (Ca^2+^), hydrogen (H^+^), and sodium (Na^+^) ions, respectively. In addition, the increased sensitivity to potassium was linearly proportional to the increase in the R_CG_:R_SG_ ratio.

Table 2 summarizes the sensitivity of the proposed potassium-selective resistively coupled coplanar-gate EGFET biosensor for various ions. Consequently, the prepared potassium-selective biosensor could not only selectively detect potassium but could also amplify the low potassium sensitivity of 51.9 mV/dec to 597.1 mV/dec, that is, by 11.5-fold, in accordance with a change in the R_CG_:R_SG_ ratio.

Table 3 presents a comparative analysis of the results of this study with those of previous studies. Previous studies on the detection of potassium ions have reported a maximum sensitivity of 77 mV/dec. However, in this study, we achieved the highest sensitivity of 597.1 mV/dec through resistance -coupling-based self-amplification.

**Figure 7:**
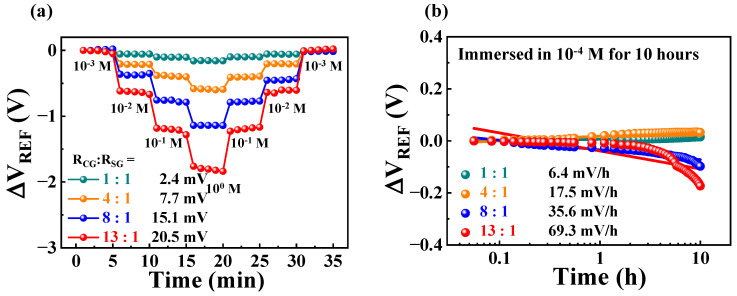
Non-ideal behaviors in potassium-sensing operations. (**a**) Hysteresis and (**b**) drift effects, at different R_CG_:R_SG_ ratios.

In the potassium-selective FET-type biosensor, the surface potential is generated by potassium transport in the sensing membrane. Repetitive sensing operations cause damage to the potassium-selective membrane, resulting in deterioration of the ion-sensing performance. Therefore, the evaluation of the hysteresis and drift effects is important to demonstrate the stability and reliability of the sensor platform. Figure 7a shows the effect of hysteresis on potassium sensing at different R_CG_:R_SG_ ratios. To evaluate the hysteresis effect, the potassium concentration of the KCl solution was changed from 10^−3^ M to 10^0^ M and then restored back to 10^−3^ M. At each potassium concentration, the transfer characteristic curve was measured five times at 1 min intervals to continuously monitor the change in V_REF_. Following that, the V_H_ was calculated by subtracting the final V_REF_ from the initial V_REF_ after 35 min of measurement. As a result, the extracted V_H_ was 2.4, 7.7, 15.1, and 20.5 mV for R_CG_:R_SG_ ratios of 1:1, 4:1, 8:1, and 13:1, respectively. Figure 7b shows the drift effect on potassium-sensing, measured after immersion in a 10^−4^ M KCl solution for 10 h. Meanwhile, the R_D_ values for potassium sensing were 6.4, 17.5, 35.6, and 69.3 mV/h for R_CG_:R_SG_ ratios of 1:1, 4:1, 8:1, and 13:1, respectively. Therefore, the introduced potassium-selective, resistively coupled coplanar-gate EGFET biosensor was verified to be a high-performance platform capable of precisely detecting potassium up to a low level of 10^−4^ M and exhibiting excellent stability and reliability even in repeated operation.

## 3. Materials and Methods

### 3.1. Materials

Glass substrate (7059 glass; Corning Inc., Corning, NY, USA); a-IGZO sputter target (In_2_O_3_:Ga_2_O_3_:ZnO = 4:2:4.1 mol%, THIFINE Co., Ltd., Incheon, Republic of Korea); ITO sputter target (In_2_O_3_:SnO_2_ = 9:1 mol%, THIFINE Co. Ltd.); SiO_2_ sputter target (purity ≥ 99.99%, THIFINE Co. Ltd.); SnO_2_ sputter target (purity ≥ 99.99%, THIFINE Co., Ltd.); 30:1 buffered oxide etchant (J.T. Baker, Phillipsburg, NJ, USA); polydimethylsiloxane (Sylgard 184 silicone elastomer; Dow corning, Midland, MI, USA); pH buffer solution (Samchun Chemical, Pyeongtaek, Republic of Korea); valinomycin (purity ≥ 98%, molecular weight = 1111.32 g/mol, Sigma-Aldrich, St. Louis, MO, USA); sodium tetraphenylborate (purity ≥ 99.5%, molecular weight = 342.22 g/mol, Sigma-Aldrich); polyvinyl chloride (purity ≥ 95%, density = 1.385 g·cm^−3^; Sigma-Aldrich); bis(2-ethylhexyl) sebacate (purity ≥ 97.0%, molecular weight = 426.67 g/mol, Sigma-Aldrich); cyclohexanone (purity ≥ 99.5%, molecular weight = 98.14 g/mol, Sigma-Aldrich); sodium chloride (NaCl, purity ≥ 99.5%, molecular weight = 58.44 g/mol, Sigma-Aldrich); calcium chloride (CaCl_2_, purity ≥ 96%, molecular weight = 110.99 g/mol, Sigma-Aldrich); potassium chloride (KCl, purity ≥ 99.0%, molecular weight = 74.55 g/mol, Sigma-Aldrich).

### 3.2. Fabrication of the Resistively Coupled TFT Transducer Unit

**Figure 8:**
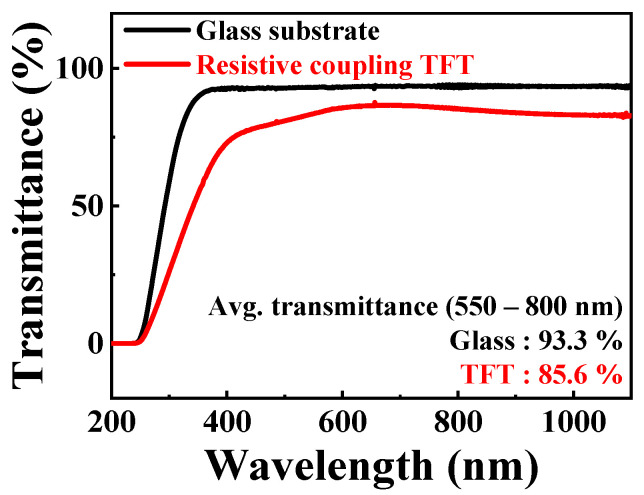
Optical transmittance spectra of the glass substrate and fabricated TFT transducer unit.

Transparent glass substrates (Corning Inc.) with dimensions of 1 × 1 cm^2^ were cleaned using a standard RCA wet-cleaning process. After depositing an ITO layer with a thickness of 150 nm on a glass substrate, coplanar gates (FG, SG, and CG) were formed on the same plane using RF magnetron sputtering and lift-off processes. Subsequently, an ITO layer with a thickness of 100 nm was deposited using RF magnetron sputtering, and resistors (R_SG_ and R_CG_) were formed by means of a lift-off process. To configure various resistances and amplification ratios, four different resistor patterns were designed, and the SG and CG were connected in series to the FG through R_SG_ and R_CG_, respectively. Then, 50 nm thick SiO_2_ and 50 nm thick a-IGZO films were deposited by means of RF magnetron sputtering as the gate oxide and channel layers, respectively. Active regions of a-IGZO TFTs with channel widths and lengths of 20 and 10 μm, respectively, were formed using photolithography and wet etching with a 30:1 buffered oxide etchant (BOE) solution. A 150 nm thick ITO film was deposited by means of RF magnetron sputtering, followed by a lift-off process to form S/D electrodes. Following that, the contact holes for the S/D electrodes were patterned using photolithography, and the SiO_2_ film was etched with a 30:1 BOE solution. Finally, we performed post-deposition annealing to improve the electrical properties of the a-IGZO TFTs in N_2_ ambient, using a furnace at 250 °C for 30 min. Figure 8 shows the optical transmittance spectra of the prepared a-IGZO coplanar-gate TFT transducer unit on a glass substrate. Under visible light (wavelength 550–800 nm), the average transmittance of the glass substrate was 93.3%, whereas that of the fabricated transducer unit was 85.6%, indicating highly transparent optical properties.

### 3.3. Fabrication of the Potassium-Selective EG-Sensing Unit

**Figure 9:**
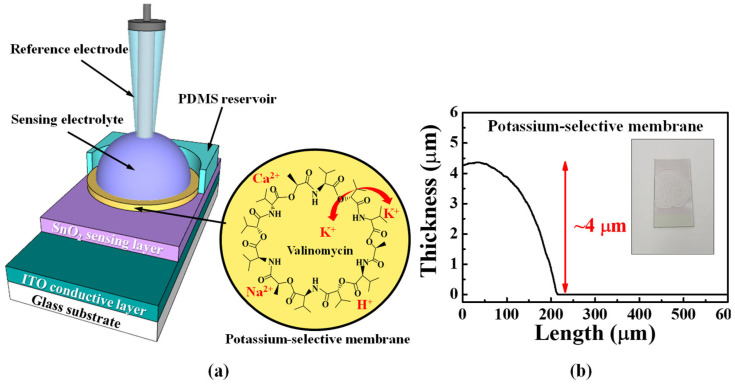
(**a**) Schematic of the potassium-selective membrane EG-sensing unit. (**b**) Thickness of the drop-cast potassium-selective membrane.

We fabricated EG-sensing units with a potassium-selective membrane on glass substrates (Corning Inc.) with a size of 1.5 × 2.5 cm^2^. The transparent glass substrates were cleaned using a standard RCA wet-cleaning process, after which a 300 nm thick ITO-conductive layer and 50 nm thick SnO_2_-sensing layer were sequentially deposited on them by means of RF magnetron sputtering. A potassium-selective membrane was formed on the SnO_2_-sensing layer by drop-casting 100 μL of the potassium ionophore mixture and drying at room temperature, for 24 h. The potassium ionophore mixture was prepared by stirring 2 mg of valinomycin (potassium ionophore), 0.5 mg of sodium tetraphenylborate (Na-TPB), 32.7 mg of polyvinyl chloride (PVC), and 64.8 mg of bis(2-ethylhexyl) sebacate (DOS) in 350 μL of cyclohexanone, at room temperature and 800 rpm, for 4 h. Valinomycin in potassium-selective membranes functions as a potassium ionophore that transports only potassium. In the stacked ITO/SnO_2_/potassium-selective membrane structure, a surface potential is generated on the SnO_2_-sensing layer and transferred directly to the SG electrode of the TFT through the ITO-conductive layer. To store the analyte and define the detection volume, we attached a polydimethylsiloxane (PDMS) reservoir with an inner diameter of 0.6 cm. Figure 9a shows the schematic of the potassium-selective membrane EG-sensing unit. The thickness of the drop-cast potassium-selective membrane was ~4 μm, as shown in Figure 9b. The inset in Figure 9b shows a photograph of the potassium-selective membrane.

### 3.4. Device Characterization

The thicknesses of the a-IGZO, ITO, SiO_2_, SnO_2_, and potassium-selective membrane layers were measured using a DektakXT Bruker stylus profiler (Bruker, Hamburg, Germany). Measurements of the electrical characteristics, including current-voltage (I-V), transfer, and output characteristics of the fabricated a-IGZO coplanar-gate TFTs were performed using an Agilent 4156B precision semiconductor parameter analyzer (Agilent Technologies, Santa Clara, CA, USA). A commercial Ag/AgCl electrode (Horiba 2086A-06T, Kyoto, Japan) was used as the reference electrode to detect the KCl, NaCl, CaCl_2_, and pH buffer solutions. Electrical measurements of the resistively coupled TFT transducer and potassium-selective biosensor platform were performed in an electromagnetically shielded dark box, to exclude external interference, such as noise, light, and contamination.

## 4. Conclusions

In this study, we propose a high-performance, potassium-selective biosensor platform. The proposed biosensor platform adopts the EGFET structure, which consists of a resistively coupled TFT transducer unit and a potassium-selective-membrane EG-sensing unit to achieve the advantages of a disposable gate electrode. The resistively coupled TFT transducer unit was fabricated transparently on a glass substrate with a-IGZO channel layers, and ITO was applied to the S/D resistive layers and gate electrodes. The average transmittance of the transparent transducer unit in the visible light range was 85.6%. The sensing unit was fabricated on the glass substrate with the ITO-conductive and SnO_2_-sensing layers to transfer the surface potential on the SnO_2_-sensing layer to the SG of the transducer unit via the ITO-conductive layer. In the transducer unit, the SG and CG were resistively coupled with the FG through resistive layers, which allowed the small surface potential biased to the SG to be amplified. By conducting pH-sensing operations, we confirmed that we could control the amplitude of the self-amplification through resistive coupling by varying and controlling the R_CG_:R_SG_ value, and the results corresponded to the results predicted by measuring the resistance of the resistive layers. In addition, the high-performance sensing characteristics of the fabricated FET-type sensor platform were verified by means of pH-sensing operations using an SnO_2_-sensing membrane. We could obtain an outstanding pH sensitivity of 712.8 mV/pH, which overcomes the physical limit on sensitivity; in addition, we ensured the stable and reliable sensing characteristics of the proposed sensor platform by measuring hysteresis and drift effects. For the selective detection of potassium, we fabricated a potassium-selective membrane on an SnO_2_-sensing layer. The potassium ionophore in the potassium-selective membrane selectively detects potassium and transfers the surface potential of the detected potassium to the SG of the transducer unit in pH, NaCl, CaCl_2_, and KCl buffers. The resistive coupling of the fabricated device linearly increased the potassium sensitivity with a change in the R_CG_:R_SG_ ratio. It was demonstrated that the biosensor exhibited remarkable sensitivity towards potassium, as evidenced by an 11.5-fold increase in potassium sensitivity from 51.9 to 597.1 mV/dec. In contrast, the sensitivity of the biosensor towards other ions such as H^+^, Ca^2+^, and Na^+^ was relatively low, with a maximum of 62.1 mV/dec. Moreover, reliability-related sensing characteristics were ensured by evaluating the hysteresis and drift effects in the KCl solution with resistive coupling. The calculated V_H_ and R_D_ had values of up to 20.5 mV and 69.3 mV/h, respectively, which were relatively small values considering the amplified potassium sensitivity. We verified the stability and reliability of the device even for repeated sensing operations. Therefore, the proposed potassium-selective FET-type biosensor, consisting of a resistively coupled TFT transducer unit and a potassium-selective EG-sensing unit, is a promising biosensor platform with high sensitivity and reliability that is capable of selective detection of potassium in an aqueous environment where various ions exist, such as blood, sweat, and food.

## Figures and Tables

**Table 1:** pH-sensing characteristics of the resistively coupled coplanar-gate EGFET sensor.

R_CG_:R_SG_	Sensitivity (mV/pH)	V_H_ (mV)	R_D_ (mV/h)	V_H_ to Sensitivity (%)	R_D_ to Sensitivity (%)
1:1	56.4	5.6	9.8	10.0	17.3
4:1	210.2	10.6	23.2	5.0	11.0
8:1	437.5	17.6	32.4	4.0	7.4
13:1	712.8	26.5	69.1	3.7	9.7

**Table 2:** Sensitivity of the potassium-selective resistively coupled coplanar-gate EGFET biosensor to various ions.

R_CG_:R_SG_	H^+^ Sensitivity (mV/pH)	Na^+^ Sensitivity (mV/dec)	Ca^2+^ Sensitivity (mV/dec)	K^+^ Sensitivity (mV/dec)
1:1	3.4	6.6	4.9	51.9
4:1	8.3	28.9	4.4	223.6
8:1	9.1	39.7	19.2	351.5
13:1	50.8	62.1	30.8	597.1

**Table 3:** Various ion-sensing performances reported in this study as well as previous studies.

Sensing Membrane	Platform	Sensitivity (mV/dec)				Reference Electrode	Test Solution	Reference
		Na^+^	K^+^	Ca^2+^	H^+^			
Al_2_O_3_	LAPS	-	50	16	43	Ag/AgCl	Tris-HCl	[34]
Al_2_O_3_	ISFET	56.9	48.1	25.7	57.2	Ag/AgCl	DI water	[35]
PS (111)	EGFET	58.76	57.9	34.9	-	Ag/AgCl	DI water	[36]
CNT	ISFET	-	62.8	26.2	-	Pt	DI water	[37]
rrPHHT	ISFET	47	77	-	-	Au	DI water	[38]
Au	ISFET	62	55	-	36	Ag/AgCl	DI water, artificial sweat	[39]
Au	EGFET	64.2	61.3	-	-	Ag/AgCl	PBS	[40]
Ti/Ag/AgCl	EGFET	38	48	36.7	-	Ti/Ag/AgCl	DI water	[41]
SnO_2_	EGFET	62.1	597.1	30.8	50.8	Ag/AgCl	DI water	This study

DI water—deionized water; PBS—phosphate-buffered saline.

## Data Availability

Not applicable.
